# Gross Appearances of the Posterior Cruciate Ligament Correlate With Its Histological Features but not With In Vivo Function in Cruciate-Retaining Total Knee Arthroplasty

**DOI:** 10.7759/cureus.38128

**Published:** 2023-04-25

**Authors:** Seiju Hayashi, Satoshi Miyazaki

**Affiliations:** 1 Orthopedic Surgery, Yawatahama City General Hospital, Ehime, JPN

**Keywords:** cruciate-retaining tka, contact point, in vivo, gross appearance, posterior cruciate ligament, total knee arthroplasty

## Abstract

Background

No study has evaluated whether the macroscopic appearance or histological features of the posterior cruciate ligament (PCL) affect the in vivo PCL function in cruciate-retaining (CR) total knee arthroplasty (TKA). The aim of this study is to elucidate the correlation between intraoperative gross appearances of the PCL and clinical parameters, their corresponding histological features, and the in vivo function.

Methods

The intraoperative gross appearances of the PCLs were evaluated; we also examined their correlations with clinical parameters, corresponding histological features, and the in vivo function in CR-TKA.

Results

There were significant correlations between intraoperative gross appearances of the PCL and that of the anterior cruciate ligament, the preoperative knee flexion angle, and the intercondylar notch stenosis. There was a significant correlation between the intraoperative gross appearance in the middle part and the corresponding histological features. However, there was no significant correlation between the intraoperative gross appearance or histological features and the PCL tension, amount of rollback, and maximum knee flexion angle.

Conclusions

The intraoperative gross appearance of the PCL correlated with clinical parameters. Also, there was a significant correlation between the intraoperative gross appearance in the middle part and the corresponding histological features; however, there was no correlation between the intraoperative gross appearance or histological features and the in vivo function.

## Introduction

The posterior cruciate ligament (PCL) is recognized as an essential stabilizer in cruciate-retaining (CR) total knee arthroplasty (TKA) [1]. Usually, the retention of the PCL is subjectively decided by a surgeon based on preoperative radiological variables or its intraoperative gross appearance [2]. However, the functional availability of the PCL is hard to determine based solely on its intraoperative gross appearance [2,3]. In CR-TKA, the PCL is retained with an assumption that it will be anatomically and biomechanically intact [4]. Since insufficiency of the degenerated PCL may affect the postoperative function of the retained PCL in CR-TKA [4], the objective criteria to predict the microscopic status of the PCL pre- or intraoperatively should be investigated.

Histopathological degenerative changes in the PCL begin early, even before articular cartilage degeneration is observed. These changes begin in the proximal part and progress to the distal part as osteoarthritis (OA) progresses [5,6]. Although several previous studies reported no significant correlation between the intraoperative gross appearance of the PCL and its histological features [7], they have not elucidated detailed intraoperative gross appearances of the PCL and their corresponding histological features. Moreover, no previous study has evaluated whether the macroscopic appearance or histological features of the PCL affect the in vivo PCL function in CR-TKA.

Therefore, we tried to define objective criteria to predict the postoperative function of the residual PCL in CR-TKA. In detail, this study aimed to (1) elucidate the preoperative and intraoperative clinical parameters correlating to the intraoperative gross appearances of the PCL, (2) compare intraoperative gross appearances in each proximal-to-distal part of the PCL and their corresponding histological features, and (3) elucidate the correlation between the intraoperative gross appearance and histological features of the PCL and the in vivo PCL function in CR-TKA.

We hypothesized that (1) intraoperative macroscopic appearances of the PCL would be predicted by preoperative and intraoperative clinical parameters, (2) intraoperative macroscopic appearances in each proximal-to-distal part of the PCL indirectly represent their corresponding histological features, and (3) intraoperative macroscopic appearances and histological features of the PCL affect postoperative the in vivo PCL function in CR-TKA.

## Materials and methods

Patients

This study was approved by the Institutional Review Board and Ethics Committee of our institute (IRB No. 20220214-001) and was conducted per the guidelines of the Helsinki Declaration. Informed consent was obtained from all individual participants. From July 2021 to April 2022, a total of 61 consecutive knees of 40 patients who underwent TKA at our hospital were prospectively enrolled in this study. Patient inclusion criterion was those who underwent primary CR-TKA based on the diagnosis of OA and osteonecrosis. The patient exclusion criteria were (1) whole-joint diseases such as rheumatoid arthritis or infections, (2) the absence of the PCL (when the ligament shape does not remain or a clear tear is observed, the PCL was judged to have disappeared), (3) the occurrence of intraoperative troubles, including fractures and ligament ruptures, and (4) the need for augmented prostheses.

Surgical technique

**Figure 1:**
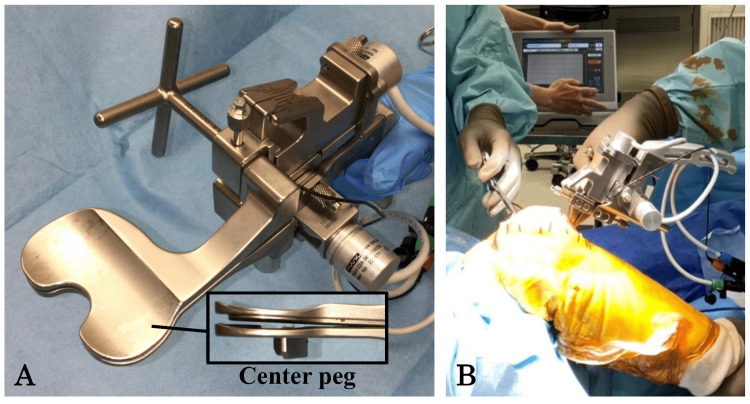
Intraoperative component gap measurement. (A) A digital knee balancer (DynAccurate, A&D, Tokyo, Japan) was used for gap measurements.  (B) Intraoperative component gap measurement.

All surgical operations were performed by a single experienced surgeon via a measured resection technique using CR-TKA (Triathlon, Stryker Orthopedics, Mahwah, NJ, USA). Longitudinal skin incisions and a medial parapatellar approach were employed. An 8-mm-thick portion of the distal femur was excised using an intramedullary device and then a 9-mm-thick portion of the proximal tibia was excised from the proximal lateral tibial bone surface, perpendicular to its axis, with a posterior slope of 5°, using an extramedullary device. The PCL was carefully protected from being cut. Any medial osteophytes, the anterior cruciate ligaments (ACLs), and both menisci were resected. The posterior bone spur was resected, and, if required, posterior capsular release was also performed. Finally, a manual distraction force was applied to the knee at full extension to confirm that a joint gap of at least 17 mm was indeed obtained. As for the femur, the size of the prosthesis was decided via a posterior referencing method using an advanced gap sizer (AGS Stryker Orthopedics), avoiding anterior notch formation. Initially, the posterior condyle was cut using a femoral cutting guide, after which a manual distraction force was applied to the knee at 90° flexion to confirm that a joint gap of at least 17 mm was indeed obtained. After all bone cutting was done and the determined size of the femoral component was set, a digital knee balancer (DynAccurate, A&D, Tokyo, Japan) was set in a patella-reduced position, and a continuous opening force was applied to measure the component gaps at 0° extension and 90° flexion (Figures 1A, 1B). The component gap at an opening force of 30 pounds was used as a measurement in this study. Finally, the thickness of the polyethylene tibial insert was decided so that the knee could obtain a complete extension, referring to the 0° extension joint gap. The “joint laxity” at 90° flexion after all component implantation was defined as the 90° flexion joint gaps minus the total thickness of the prosthesis (i.e., femoral component, tibial tray, and tibial insert).

Preoperative clinical features

The preoperative clinical parameters of each patient, including the age and range of motion (ROM) of the knee, were evaluated. The Kellgren-Lawrence (K/L) classification [7] and the femoro-tibial angle (FTA), measured on a standing weight-bearing anteroposterior radiograph, were also evaluated.

Intraoperative assessment

**Figure 2:**
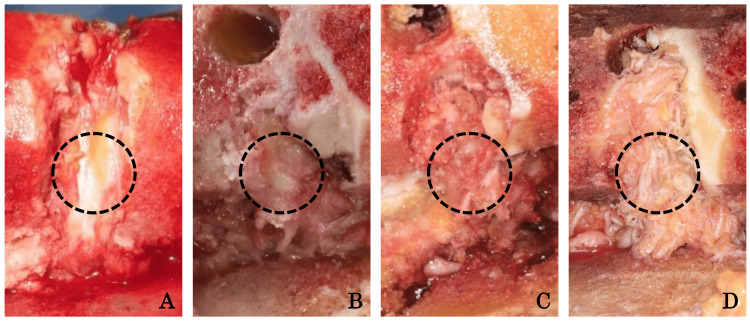
Evaluation of intraoperative gross appearance of the PCL. (A) Intact (0 points), (B) mildly degenerated (1 point), (C) moderately degenerated (2 points), and (D) severely degenerated (3 points). A black dotted circle showed an example of the evaluated lesion. PCL, posterior cruciate ligament

The intercondylar notch stenosis was evaluated, and the degree of stenosis was graded from 0 to 3 points according to the occupation rate (<25%, 25%-50%, 51%-75%, and >75%, respectively) [8]. The intraoperative gross appearance of the ACL was graded from 0 to 3 points (normal, frayed, partially ruptured, and absent, respectively) [8]. The whole length of the PCL was evenly divided into three parts (proximal, middle, and distal parts), and their intraoperative gross appearances were also graded from 0 to 3 points (intact, mildly degenerated [roughed surface], moderately degenerated [frayed], and severely degenerated [partially ruptured], respectively) at each part (Figures 2A-2D).

Radiological assessment under anesthesia

**Figure 3:**
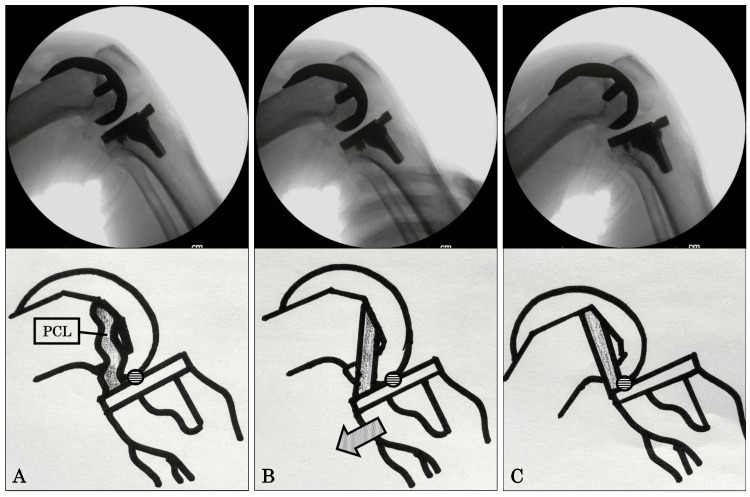
Radiological assessment. Upper columns show images of fluoroscopy, and lower columns show their schematic diagrams. (A) Gravity sag, (B) posterior stress, and (C) maximum knee flexion view. A circle showed contact point. An arrow showed direction of the posterior stress.

**Figure 4:**
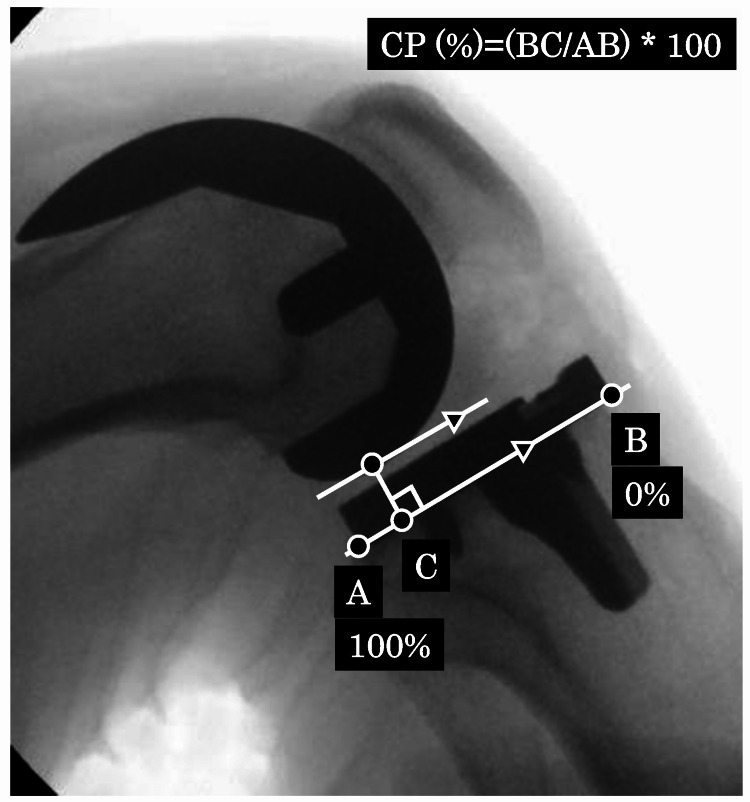
Evaluation of the CP. The CP (CP (%) = (BC/AB) × 100) was calculated. The distance from the anterior edge of the tibial component to the CP (lines B–C) divided by the width of the tibial component (lines A–B). CP, contact point

Immediately after surgery, fluoroscopy was performed under anesthesia. First, an image (gravity sag view) was obtained at 90° flexion in the supine position (Figure 3A). Then, another image (posterior stress view) was obtained via the application of maximal manual posterior stress to the tibia at 90° flexion by the surgeon (Figure 3B). Finally, an image of the maximum knee flexion view was obtained (Figure 3C). The contact point (CP; CP (%) = (BC/AB) × 100) in each obtained view was calculated as the distance from the anterior edge of the tibial component to the tibiofemoral CP (Figure 4, lines B-C) divided by the total width of the tibial component (Figure 4, lines A-B) to indirectly evaluate the PCL function. The anterior edge of the tibial component was defined as 0% and the posterior edge was defined as 100%. Each measurement value was defined as either sag-CP, stress-CP, or flexion-CP (Figures 3A-3C). All image evaluations were performed using a computer software program (Synapse® PACS). The “PCL tension” was defined as the distance covered for the PCL to become tense, which was calculated by subtracting the sag-CP from stress-CP. The higher the tension of the PCL, the less the distance moved, and the lower the tension of the PCL, the more the distance moved. The “amount of rollback” was defined as the distance calculated by subtracting the stress-CP from the flexion-CP (Figures 3B, 3C). Each component alignment angle (α, β，γ，δ angle) was also measured on postoperative X-rays [9].

Histological assessment

**Figure 5:**
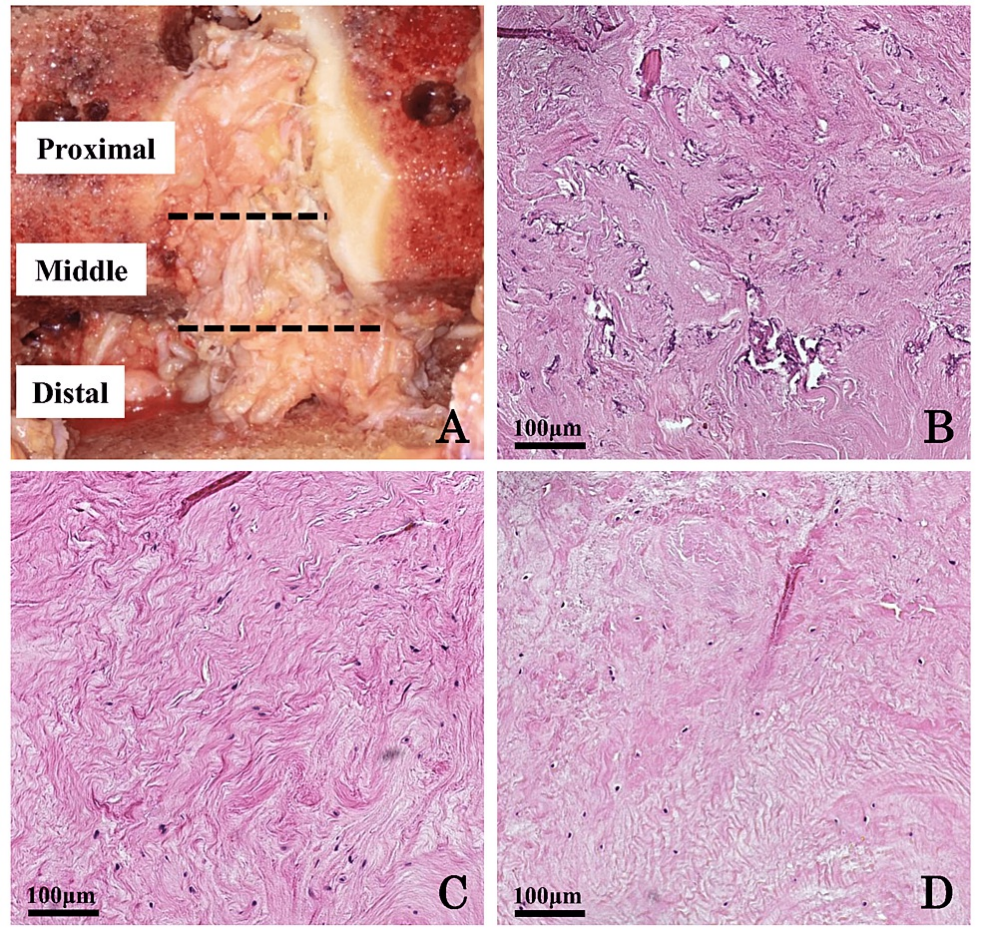
Histological evaluation of the PCL. (A) A piece of the PCL tissue excised from each part (proximal, middle, and distal parts) was stained with hematoxylin-eosin for histological evaluations using modified histological scoring: (B) proximal part, (C) middle part, and (D) distal part (×100). PCL, posterior cruciate ligament

The whole length of the PCL was evenly divided into three parts (proximal, middle, and distal parts; Figure 5A). A small slit was made in each part, and a piece of ligament tissue measuring 1 × 1 × 1 mm was excised from the center of the parenchyma. Then, it was fixed in 4% paraformaldehyde phosphate-buffered saline (ASIAKIZAI Inc., Tokyo, Japan), embedded in paraffin, and cut into 4-μm-thick sections. Each specimen was stained with hematoxylin-eosin for histological evaluations (Figures 5B-5D). At least two randomized regions from each part were histologically evaluated under a high-power field (×100), and the average values of the histological scores were used for statistical analyses. A modified semiquantitative scale was used for histological evaluations [5,6]. Each subscale (inflammation in the ligament substance, mucoid degeneration, chondroid metaplasia, cystic changes, and orientation of collagen fibers) was graded from 0 to 3 points (normal, mild degeneration, moderate degeneration, and severe degeneration, respectively). Two blinded investigators individually assessed each sample.

Statistical analysis

The results of this study are expressed as the mean ± standard deviation (SD). Data were analyzed using EZR [10]. Spearman’s rank correlation analysis was used to assess the correlation between preoperative or intraoperative clinical parameters and the intraoperative macroscopic appearance, and between intraoperative macroscopic appearances of the PCL and its histological features. Spearman’s rank correlation analysis was also used to assess the correlation between the intraoperative macroscopic appearance or the histological features of the PCL and its tension, the amount of rollback, and the postoperative deep knee flexion angle. The reliability of all evaluations was confirmed by examining the intra- and interobserver reliabilities using the interclass correlation coefficient (ICC). P < 0.05 was considered statistically significant for all analyses.

## Results

Seven knees in six patients were subsequently excluded from the analysis because they did not meet the inclusion criteria. Finally, a total of 54 knees in 34 patients were retained for data analyses. Patients’ preoperative and intraoperative demographic data are summarized in Table 1. Postoperative measurement values are summarized in Table 2. Intraoperative gross appearances of the PCL and their histological scores are summarized in Table 3.

**Table 1:** Preoperative patient characteristics and intraoperative measurement values. BMI, body mass index; FTA, femorotibial angle; K/L, Kellgren-Lawrence; ACL, anterior cruciate ligament

Measurement values	Mean ± SD	Range (median)
Age (y)	82 ± 9	59–98 (81.5)
Height (m)	1.5 ± 0.1	1.35–1.72 (1.5)
Body weight (kg)	60.5 ± 13.2	39.0–97.0 (58.0)
BMI (kg/m^2^)	25.9 ± 5.4	17.3–45.5 (25.2)
FTA (°)	183.9 ± 6.5	163.0–193.0 (185.0)
Preoperative extension (°)	-12.8 ± 10.7	-45.0 to 0 (-10.0)
Preoperative flexion (°)	120.5 ± 15.1	75.0–145.0 (120.0)
Extension component gap (mm)	8.9 ± 1.2	7.5–13.3 (8.8)
Flexion component gap (mm)	11.3 ± 2.2	8.5–18.3 (10.7)
Joint laxity (mm)	1.4 ± 1.8	-2.2 to 8.3 (1.03)
K/L classification (0/1/2/3/4)	0/0/4/32/18	-
Gross appearance of ACL (0/1/2/3)	4/20/12/18	-
Notch stenosis (0/1/2/3)	15/28/10/1	-

**Table 2:** Postoperative measurement values. CP, contact point

Measurement values	Mean ± SD	Range (median)
Component alignment angle		
α (°)	95.5 ± 1.7	92.0 -100.0 (95.5)
β (°)	88.9 ± 1.3	85.0–92.5 (89.0)
γ (°)	2.2 ± 2.4	-3.0 to 14.0 (1.5)
Δ (°)	84.8 ± 2.2	79.5–89.0 (84.8)
Postoperative flexion (°)	119.8 ± 7.7	99.5 -131.5 (121.5)
Sag-CP (%)	68.3 ± 5.7	57.0–79.7 (68.6)
Stress-CP (%)	62.1 ± 7.6	46.3–81.5 (62.4)
Flexion-CP (%)	76.0 ± 6.6	56.6–89.0 (76.6)
PCL tension	6.2 ± 4.5	-2.5 to 17.0 (6.1)
Amount of rollback	13.8 ± 7.8	-4.5 to 33.1 (12.9)

**Table 3:** Intraoperative gross appearance in each proximal-to-distal part of the PCL and its histological appearance and scoring. PCL, posterior cruciate ligament

Evaluations	Proximal	Middle	Distal
Gross appearance	1.7 ± 0.8	1.4 ± 0.7	1.2 ± 0.7
Histological appearance	1.2 ± 1.2	0.5 ± 0.8	0.7 ± 0.9
Inflammation	1.8 ± 0.9	1.4 ± 0.8	1.2 ± 0.8
Mucoid degeneration	1.4 ± 0.8	1.0 ± 0.7	0.8 ± 0.7
Cystic changes	1.0 ± 1.0	0.5 ± 0.8	0.8 ± 1.0
Orientation of collagen fibers	1.2 ± 1.0	0.6 ± 0.8	0.7 ± 0.8
Total histological score	6.6 ± 2.8	4.0 ± 2.5	4.2 ± 2.8

**Figure 6:**
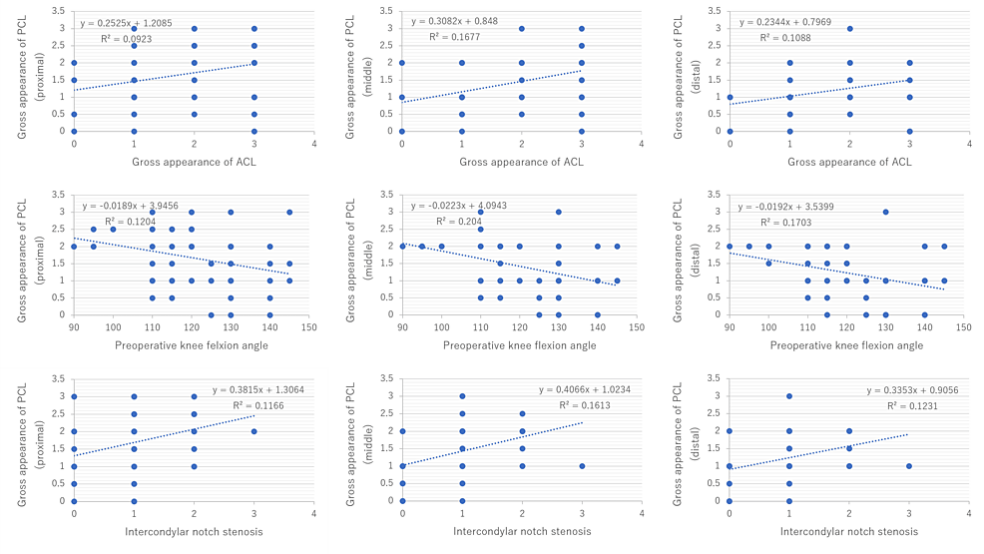
Correlations between the intraoperative gross appearance of the PCL and the intraoperative gross appearance of the ACL, preoperative knee flexion angle, and the intercondylar notch stenosis. PCL, posterior cruciate ligament; ACL, anterior cruciate ligament

There were significant correlations between the intraoperative gross appearance in each proximal-to-distal part of the PCL and the intraoperative gross appearance of the ACL (r = 0.31, P = 0.02; r = 0.41, P < 0.01; and r = 0.35, P < 0.01, respectively), preoperative knee flexion angle (r = −0.32, P = 0.02; r = −0.45, P < 0.01; and r = −0.43, P < 0.01, respectively), and the intercondylar notch stenosis (r = 0.37, P < 0.01; r = 0.45, P < 0.01; and r = 0.39, P < 0.01, respectively) (Figure 6). There was no significant correlation between the intraoperative gross appearance in each proximal-to-distal part of the PCL and age (r = 0.15, P = 0.29; r = 0.07, P = 0.63; and r = 0.05, P = 0.72, respectively), FTA (r = 0.26, P = 0.06; r = 0.25, P =0.07; and r = 0.17, P = 0.23, respectively), and K/L classification (r = 0.24, P = 0.08; r = 0.19, P = 0.16; and r = 0.22, P = 0.12, respectively).

**Figure 7:**
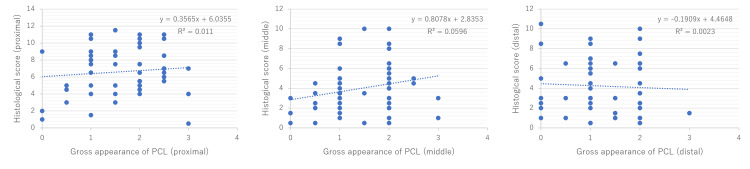
Correlation between the intraoperative gross appearance of the PCL and its corresponding histological features. PCL, posterior cruciate ligament

There was a significant correlation between the intraoperative gross appearance in the middle part of the PCL and its corresponding histological features (r = 0.28, P = 0.04). On the other hand, there was no significant correlation between the intraoperative gross appearance in each proximal and distal part of the PCL and its corresponding histological features (r = 0.07, P = 0.60; and r = −0.03, P = 0.85, respectively) (Figure 7).

There was no significant correlation between either the intraoperative gross appearance or its histological features in the middle part of the PCL and its tension (r = 0.16, P = 0.26; r = 0.04, P = 0.78, respectively), the amount of rollback (r = 0.15, P = 0.29; r = 0.05, P = 0.74, respectively), and the postoperative deep knee flexion angle (r = −0.01, P = 0.94; r = −0.06, P = 0.65, respectively).

The intra- and interobserver measurement reliabilities were good for all variables (ICC > 0.7, range: 0.75-0.97).

## Discussion

The current study revealed that (1) the intraoperative gross appearance in each proximal-to-distal part of the PCL correlated with the preoperative flexion angle, intercondylar notch stenosis, and the intraoperative gross appearance of the ACL, (2) there was a significant correlation between the intraoperative gross appearance only in the middle part of the PCL and corresponding histological features, and (3) there was no significant correlation between either the intraoperative gross appearance or histological features in the middle part of the PCL and the in vivo function of the PCL in CR-TKA.

As OA progressed, the ROM of the knee would be restricted [11]. Reactive bone formation in the intercondylar notch causes the narrowing of the notch, leading to ACL damage [12]. Radiologically higher grades of OA are likely to have a severely degenerated ACL, and the histological appearance of the PCL was associated with either the macroscopic or microscopic appearance of the ACL [3,13,14]. In this study, parameters such as the preoperative flexion angle, intercondylar notch stenosis, and the appearance of the ACL, which are easily recognized by the surgeon, were found to have significant correlations with the intraoperative gross appearance of the PCL. When surgeons perform TKA, the decision of whether or not to resect or retain the PCL is made preoperatively or based on the intraoperative clinical parameters [2,15]. However, several previous reports have demonstrated that neither preoperative and intraoperative clinical parameters nor the gross appearance of the PCL could reliably predict the extent of its histological degenerative changes [7,13,14,16-18]. Our results were almost consistent with previous reports, except that there was a significant correlation between the intraoperative gross appearance in the middle part of the PCL and the corresponding histological features. Friction from repetitive knee motions at the point of crossing with the ACL might have caused the degenerative gross appearance and histological degeneration.

Some surgeons advocate for its excision and substitution, while others recommend its preservation, selectively or systematically, arguing that it is associated with a better outcome [14]. The proponents of retaining the PCL claim that this system enables experts to maintain better knee proprioception [17,19,20], maintain ROM [21], prevent the posterior subluxation of the tibia [19,21-23], and reduce loosening and excessive polywear [24]. The choice of a CR-TKA is based on the surgeon’s assumption that the PCL is anatomically and biomechanically normal [25]. However, it is still not clear whether the degeneration of the retained PCL leads to functional insufficiency [14]. Several previous studies have shown that the PCL in OA-affected knees is affected by microscopic changes or the disorganization of collagen fibers that may be macroscopically undetected [7,13,16,18]. An arthritic ligament is less strong and stiff than a normal one [26]. These histological features would be an indirect way of assessing the biomechanical function [27]. The variable rate of late instability found in CR TKA might be explained by such morphologically intact but histologically degenerated PCL in OA-affected knees [26,28].

So far, it has not been clearly reported how the “PCL tension,” a well-known indicator of the PCL function, influences the postoperative kinematics in vivo in CR-TKA [26]. A loose PCL would induce a paradoxical roll-forward, which leads to joint instability, pain, and a restriction of the postoperative knee flexion angle [22,23,26]. On the contrary, a tighter PCL might prevent paradoxical motion, while excess tension can also limit knee flexion [22,23]. According to our results, there was no significant correlation between either the intraoperative gross appearance or histological features in the middle part of the PCL, and its tension, the amount of rollback, and the postoperative deep knee flexion angle. Hence, it is difficult to predict the postoperative in vivo PCL function in CR-TKA from the intraoperative gross appearance or histological features of the PCL. One possible explanation for these findings is that the CR-TKA prosthetic designs used in the current study may require neither a macroscopically or histologically intact ligament nor PCL tension. Although PCL insufficiency is a contraindication to CR-TKA [4], our results should be noted by surgeons.

Several limitations of this study should be noted. First, all parameters that were measured under anesthesia may not reflect the awakened situation [26]. However, excluding the muscle resistance force enabled us to determine the simple joint function. Second, the specimens used for histological assessments do not truly reflect whole regions of the PCL. However, the histological evaluations were performed with several randomized regions, and the average values were used for evaluations to minimize technical errors. Third, the sample collection might affect postoperative in vivo PCL function. However, 1 × 1 × 1 mm size is very small, and the impact would be minimal. Fourth, the geometry of the prosthesis would affect the results. Whether highly conforming inserts could fill in for the PCL function remains controversial [29], and different results would be obtained when using other prostheses. Moreover, postoperative deep knee flexion angle would be affected not only by prosthesis design but also by posterior tibial slope (δ angle) or “joint laxity”. Although data were not shown in the manuscript, there was no correlation between the δ angle or “joint laxity” and postoperative deep knee flexion angle. Finally, the correlation between those macroscopical or histological evaluations and the in vivo PCL function in CR-TKA’s effect on long-term clinical outcomes remains unknown. Longer-term follow-up evaluations are needed for further investigations.

## Conclusions

The current study revealed that the intraoperative gross appearance in each proximal-to-distal part of the PCL correlated with the preoperative flexion angle, intercondylar notch stenosis, and the intraoperative gross appearance of the ACL. Also, there was a significant correlation between the intraoperative gross appearance only in the middle part of the PCL and corresponding histological features; however, there was no significant correlation between either the intraoperative gross appearance or histological features in the middle part of the PCL and the in vivo function of the PCL in CR-TKA. Hence, it was difficult to predict the postoperative in vivo function of the residual PCL in CR-TKA from its pre- or intraoperative clinical parameters.
